# Use of HLA desensitization in the management of renal transplant recipients in Europe

**DOI:** 10.3389/fimmu.2025.1451135

**Published:** 2025-08-28

**Authors:** Lionel P. E. Rostaing, Georg A. Böhmig, Muhammed Mahdi Taqi, Ben Gibbons

**Affiliations:** ^1^ Service de Nephrologie, Hemodialyse, Apherèses et Transplantation Renale, Centre Hospitalier Universitaire de Grenoble, Grenoble, France; ^2^ Clinical Department of Nephrology and Dialysis, University Clinic for Internal Medicine III, Medical University of Vienna, Vienna, Austria; ^3^ Bryter Inc., New York, NY, United States

**Keywords:** desensitization, human leukocyte antigen (HLA), kidney transplantation, donor-specific antibody (DSA), antibodies, anti-HLA antibody

## Abstract

A significant challenge in kidney transplantation is overcoming immunological barriers such as human leukocyte antigen (HLA) incompatibilities. The presence of anti-HLA antibodies in the transplant candidate is referred to as HLA sensitization. As the degree of sensitization increases, the likelihood of finding a compatible organ decreases. Desensitization is the process of reducing recipient anti-HLA antibodies to acceptable levels to allow transplantation. Enthusiasm for the process has declined with focus turning to optimizing deceased donor allocation and paired kidney exchange programs. This research was designed to assess current practices around desensitization in Europe. A 15-minute online survey with 56 multiple choice or open-ended questions was completed by EU transplant nephrologists, transplant surgeons and nephrologists. Survey topics included kidney transplant caseloads, pre-transplant desensitization and desensitization post-transplant. The results indicate high variation in what physicians consider to be the threshold mean fluorescence intensity (MFI) level for significant anti-HLA antibodies and the need for desensitization. Desensitization protocols are not standardized; while there is alignment on the usage of apheresis and IVIG, usage of other agents is variable. New therapies for desensitization are emerging which could aid in removing immunological barriers to transplantation for the most highly-sensitized patients.

## Introduction

Kidney transplantation is the optimal treatment route for end-stage kidney disease (ESKD) patients. It is associated with improved survival and quality of life compared with chronic hemodialysis (1, 2). While transplant rates are increasing, several challenges must be addressed to improve outcomes. These include overcoming immunological barriers between donor and recipient, such as blood group and human leukocyte antigen (HLA) incompatibilities.

Mismatching of HLA between donor and recipient leads to the production of anti-HLA antibodies by the recipient against the donor organ. The presence of anti-HLA antibodies in the prospective recipient is referred to as HLA sensitization. Sensitization typically occurs via blood products or previous transplantation; however, ‘spontaneous’ sensitization has been reported wherein patients possess anti-HLA antibodies in the absence of any known exposure (3, 4).

Sensitization can be measured using a range of tests; complement-dependent cytotoxicity (CDC) testing where sensitization is expressed as a proportion of panel reactive antibodies (%PRA), flow cytometric crossmatch (FCM) with results measured by channel shift and expressed as a positive or negative crossmatch to an antigen, and solid phase assays using single antigen beads with results expressed as Mean Fluorescence Intensity (MFI). General practice in Europe for anti-HLA antibody detection involves solid phase antibody detection assays with results expressed as MFI. There is no universal agreement on MFI thresholds across Europe; centers use thresholds between 1000 and 3000, but the exact values depend on local practices and the clinical context (10). Most centers use MFI values of 1,000–1,500 as significant with a minority starting at 500 (10, 17), and MFI values above 3000 are generally associated with a higher risk of antibody-mediated rejection (10, 17, 38).

HLA antibody testing using single antigen bead technology uses the One Lambda and Immucor techniques. These two techniques employ similar principles but often yield differing results due to variations in bead design, antigen density, and detection thresholds (38–40). Studies have demonstrated that One Lambda tends to exhibit higher sensitivity, potentially detecting low-level antibodies that may not be clinically relevant, whereas Immucor may provide a more specific profile by reducing false-positive results (38–40). These differences can lead to discrepancies in the identification of unacceptable antigens and calculated panel reactive antibody values.

The presence of pre-formed HLA antibodies is associated with rejection of the renal allograft (5, 6), and the level of HLA mismatching is correlated with transplant outcomes in terms of survival and rejection rates (7, 8). Around 30% of patients on renal transplant waiting lists are considered sensitized (1, 9, 37) or highly sensitized (defined as percentage of panel reactive antibody (%PRA) of >85% (10) or calculated panel reactive antibody (cPRA) of >80% (11, 37)). As the degree of sensitization increases, the likelihood of finding a compatible organ decreases (9). Highly sensitized patients are likely to spend longer on transplant waiting lists (4, 6) due to the severity of their sensitization, which may in turn impact their post-transplant outcomes (12).

Desensitization protocols have been in use for over two decades, with the goal of removing or reducing recipient’s anti-HLA antibodies targeting donor organ. This process allows transplantation across immunological barriers, increasing the availability of donor organs to sensitized patients. Vo et al. (2013) (13) demonstrated the potential benefit of desensitization for highly sensitized patients who may otherwise be unable to receive a transplant. Their results showed patients who received an incompatible allograft following desensitization with IVIG and rituximab had a survival benefit over those who solely remained on dialysis. While desensitization has increased transplant rates for sensitized patients, its popularity has declined over recent years. Desensitizing protocols are expensive, resource intensive and expose patients to a greater risk of morbidities, such as severe infections, due to additional immunosuppression (1, 2). Paired donation schemes/kidney exchange programs (an exchange involving two or more incompatible pairs who swap their donors to achieve a compatible transplant for both recipients) and the prioritization of highly sensitized and sensitized patients in kidney allocation programs (deceased donor kidneys) have provided an alternative route to overcoming immunological incompatibility and have increased transplant rates in sensitized patients. Patients undergoing HLA-incompatible transplantation following desensitization (using a plasmapheresis and IVIG protocol for example) have been shown to experience poorer long-term outcomes in terms of survival, graft survival and histological damage to the kidney allograft than those receiving a HLA-compatible allograft (1, 5, 9). Therefore, in countries where it is possible, entry into a kidney exchange program is the preferred choice over desensitization (10) for the management of patients with HLA antibodies, and is recommended by The European Society for Organ Transplantation’s most recent consensus guidelines. Furthermore, Manook et al. (2017) (36) demonstrated patient survival is the same with dialysis compared to HLA incompatible kidney transplantation, further contributing to the decline in popularity of the desensitization protocol.

Despite their relatively long history of use, desensitization protocols are not standardized and vary considerably by center. Current desensitization protocols involve removing/reducing levels of anti-HLA antibodies prior to transplantation, and then again immediately following transplantation. IVIG, apheresis, anti-CD20 monoclonal antibodies (e.g., rituximab), proteasome inhibitors (e.g., bortezomib) and the anti-C5 monoclonal antibody eculizumab (2, 10) are therapies used in current protocols. Rounds of plasma exchange/immunoadsorption and IVIG or anti-CD20 monoclonal antibodies (10) prior to transplantation are the most frequently used protocols with the most compelling evidence to date. Induction therapies such as anti-thymocyte globulin may also be used at the time of transplant to reduce the chance of immunological rejection.

Despite the risks associated with desensitization, for some patients, it may still be the best choice. Individual patient factors such as level of sensitization, the predicted time on waiting lists, likelihood of finding a HLA compatible deceased or living donor, and the chances of success and/or complications are all items physicians must consider before proceeding to desensitization.

This research was designed to assess current practices around desensitization in Europe, from understanding how pre-sensitized patients are identified, to current approaches to desensitization pre-transplant and post-transplant.

## Methodology

Forty transplant nephrologists, transplant immunologists, transplant surgeons, nephrologists and immunologists were recruited through targeted lists from specialist panels, then screened and profiled to ensure a valid representation of the European market (CONSORT in **Supplementary Material**). The study ran from February to November 2022. In order to qualify for the study, physicians must have performed kidney transplants in 2020 or 2021, must have been practicing for 3–30 years, must perform DSA testing, and must be involved in the treatment of acute antibody mediated rejection. In addition to study-specific screening criteria, respondents were screened to ensure that they are not affiliated with any industry partners. A full breakdown of sample demographics is shown in **Table 1**.

**Table 1:** Table showing respondent profile breakdown (N=40).

Demographics		Total (n)	Total (%)
All respondents		40	100
Specialty	Transplant nephrologist	24	60
	Transplant surgeon	11	27.5
	Nephrologist	3	7.5
	Transplant Immunologist	1	2.5
	Immunologist	1	2.5
Gender	Male	27	67.5
	Female	12	30
	Not specified	1	2.5
Length of time in practice	3–10 years	11	27.5
	11–20 years	14	35
	21+	15	37.5
Hospital type	Teaching/university hospital	30	75
	General hospital	6	15
	Private hospital	2	5
	Not specified	2	5
Country of practice	Italy	6	15
	United Kingdom	5	12.5
	France	4	10
	Spain	4	10
	Croatia	3	7.5
	Poland	2	5
	Portugal	2	5
	Turkey	2	5
	Albania	1	2.5
	Austria	1	2.5
	Belgium	1	2.5
	Czech Republic	1	2.5
	Denmark	1	2.5
	Germany	1	2.5
	Greece	1	2.5
	Netherlands	1	2.5
	Switzerland	1	2.5
	Ukraine	1	2.5
	Romania	1	2.5
	Montenegro	1	2.5

A 15-minute online survey was completed with 56 questions that were grouped into sections: kidney transplant caseloads, pre-transplantation desensitization, desensitization post-transplantation, and demographic questions. The survey included a range of different question types, including: dichotomous (yes/no) questions, pre-coded (multiple choice) questions, and open-ended responses. A copy of the survey instrument is provided as **Supplementary Information**.

Data was aggregated and described using the mean and range. In order to determine whether findings were statistically significant, t-test for quantitative or continuous and chi-squared for categorical data were used. A p-value ≤0.05 was considered statistically significant.

## Results

### Kidney transplant caseloads

In 2020, an average of 65 renal transplants were performed by respondent physicians. A significant increase was seen in 2021, where an average of 74 renal transplants were performed (t(39) = 3.04, *p* =.004). Of those on the waiting list to receive a transplant in 2020 and 2021, an average of 18% and 19% were highly sensitized candidates (cPRA >80%).

Luminex^®^ platform assays are the most commonly performed test by physicians (88%) to detect anti-HLA antibodies in potential kidney transplant recipients. They are significantly more likely to be using the One Lambda platform than the Immucor platform (71% currently use One Lambda, vs. 34% Immucor, t(34) = 2.61, *p* = .01).

**Figure 1:**
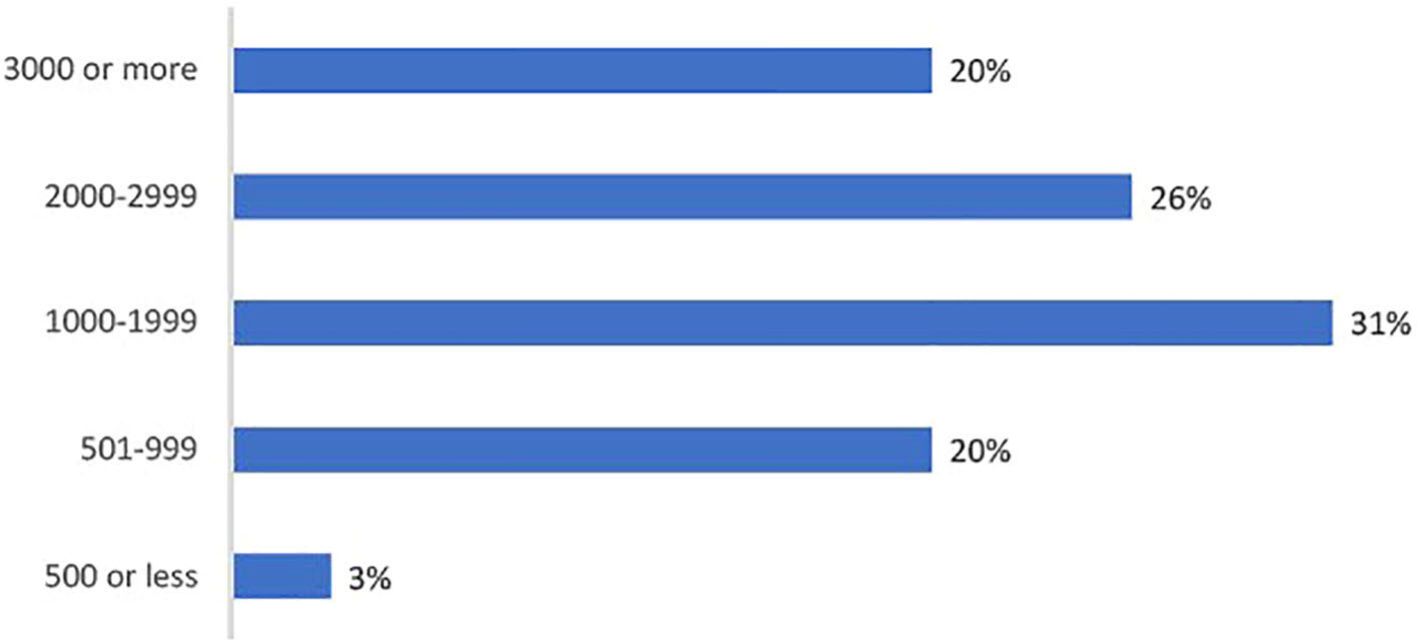
Bar charts showing percentage indicating the MFI threshold above which physicians would consider anti-HLA antibodies to be significant (n=35).

Physicians were not unified in what they considered to be the mean fluorescence intensity (MFI) threshold above which they would consider anti-HLA antibodies significant. Nearly all (97%) indicated that it would need to be greater than 500. However, values greater than 500 varied, with 20% indicating it would need to be between 501-999, 31% indicating between 1000-1999, 26% indicating between 2000–2999 and 20% indicating 3000 or greater (see **Figure 1** for full breakdown).

### Desensitization protocols prior to transplant

**Figure 2:**
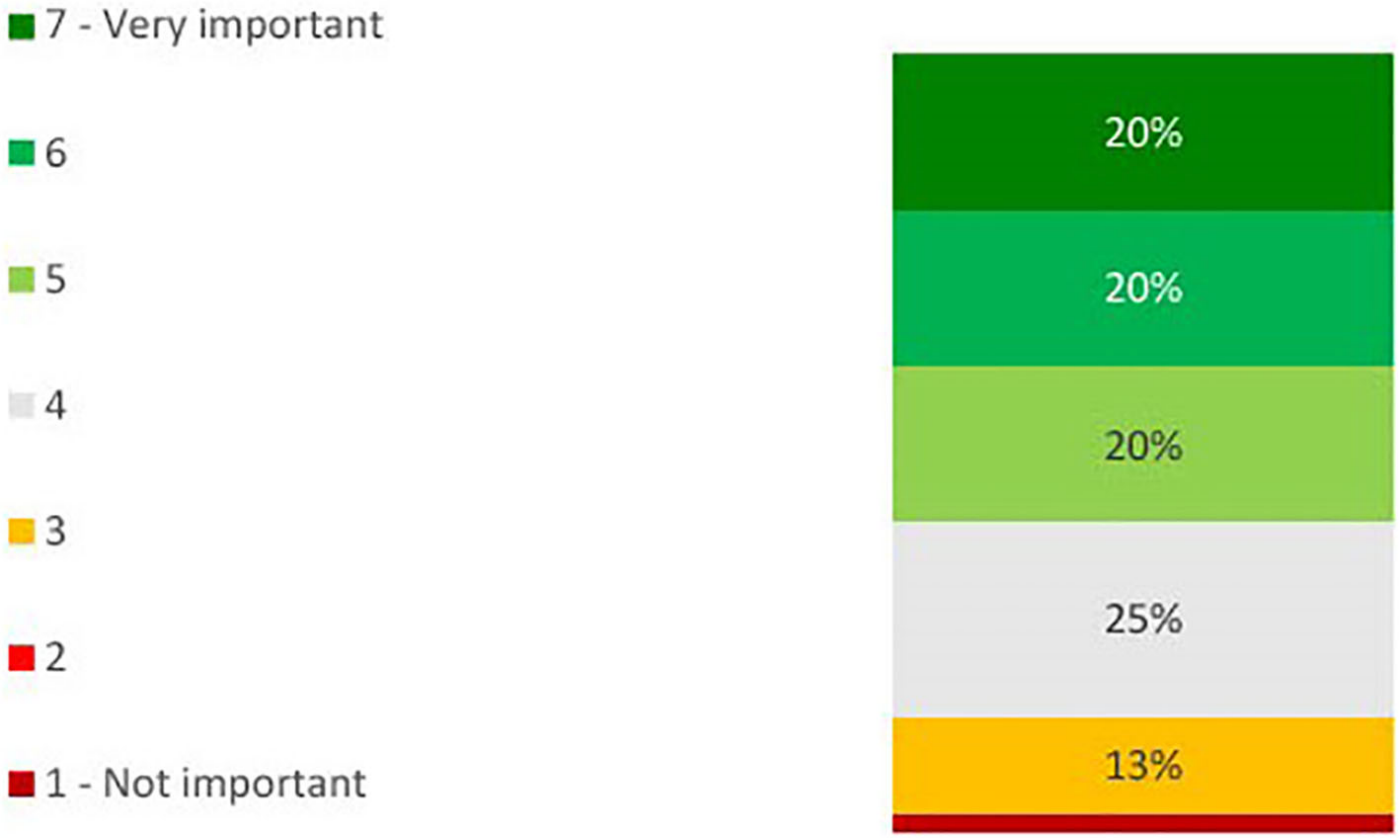
Bar chart showing percentage physicians indicating the level of importance of desensitizing kidney transplant candidates on a scale of 1 to 7, where 1 is ‘not at all important’ and 7 is ‘very important’ (n=40).

**Figure 3:**
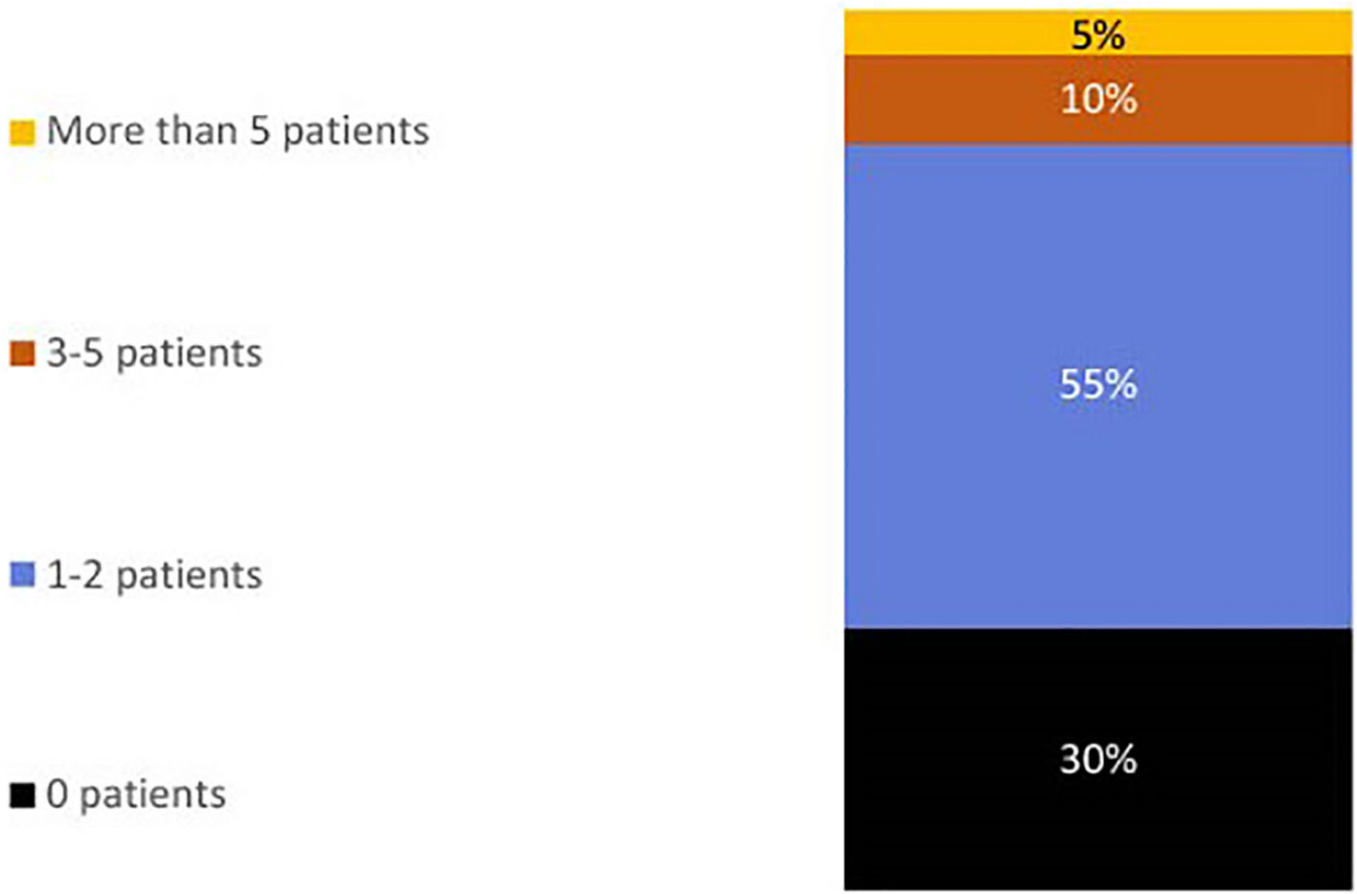
Bar chart showing percentage physicians indicating the number of kidney transplant recipients they desensitize pre-transplant per year (n=40).

On the importance of desensitization of kidney transplant recipients, physicians’ views were mixed (see **Figure 2**). On a scale of 1 to 7, where 1 is not important and 7 is very important, 40% gave a score of 6 or 7. The number of kidney transplant recipients who are desensitized each year varied by physician (see **Figure 3**). 70% desensitize at least 1 candidate per year, with 65% desensitizing 1 to 5 patients.

**Figure 4:**
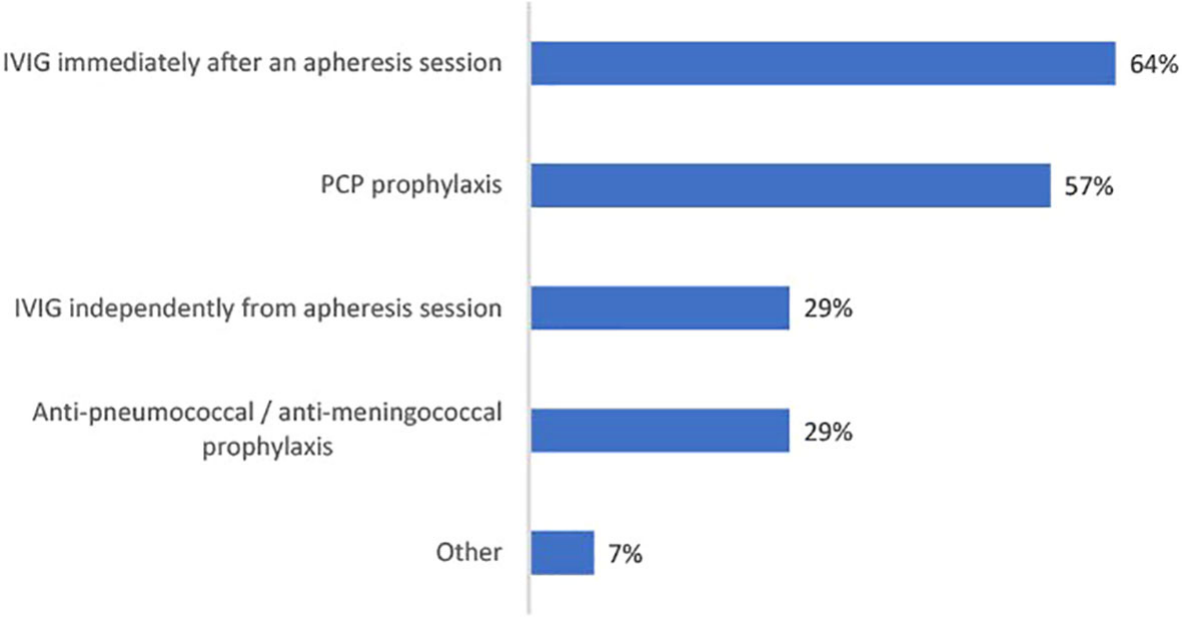
Bar chart showing percentage physicians using each pretransplant prophylactic therapy as part of their desensitization protocol (n=28).

Among those who desensitize at least 1 candidate a year, nearly all (93%) are using apheresis, mainly because it’s a mandatory part of their desensitization protocol. Apheresis was mandatory for 77% who had apheresis in their current desensitization protocol. In cases where apheresis is used, it is based on plasmapheresis (88%), double-filtration plasmapheresis (19%), semi-specific adsorption (27%). Further, 12% would use these approaches in combination. Plasmapheresis is significantly more likely to be used than double-filtration plasmapheresis (t(25) = 6.43, *p* <.001) or semi-specific adsorption (t(25) = 4.50, *p* <.001). Immediately after an apheresis session, IVIG is prescribed by 64% of physicians, while 29% prescribe IVIG independently from apheresis (either prescribing apheresis and IVIG separately, or IVIG without apheresis) (**Figure 4**). The dosage of IVIG prescribed varies. 14% prescribe a dose of IVIG less than 0.5g/kg, 48% prescribe between 0.5 to 1g/kg, while 38% prescribe more than 1g/kg. Prophylactic therapies are prescribed against Pneumocystis pneumonia (PCP) by 57% of physicians. Anti-pneumococcal/anti-meningococcal prophylaxis is used by 29% of physicians.

Antithymocyte globulin (ATG) and basiliximab are the induction therapies used at transplantation. 68% of physicians will use ATG, and 58% of physicians will use basiliximab.

### Prophylactic desensitization protocols post-transplant

**Figure 5:**
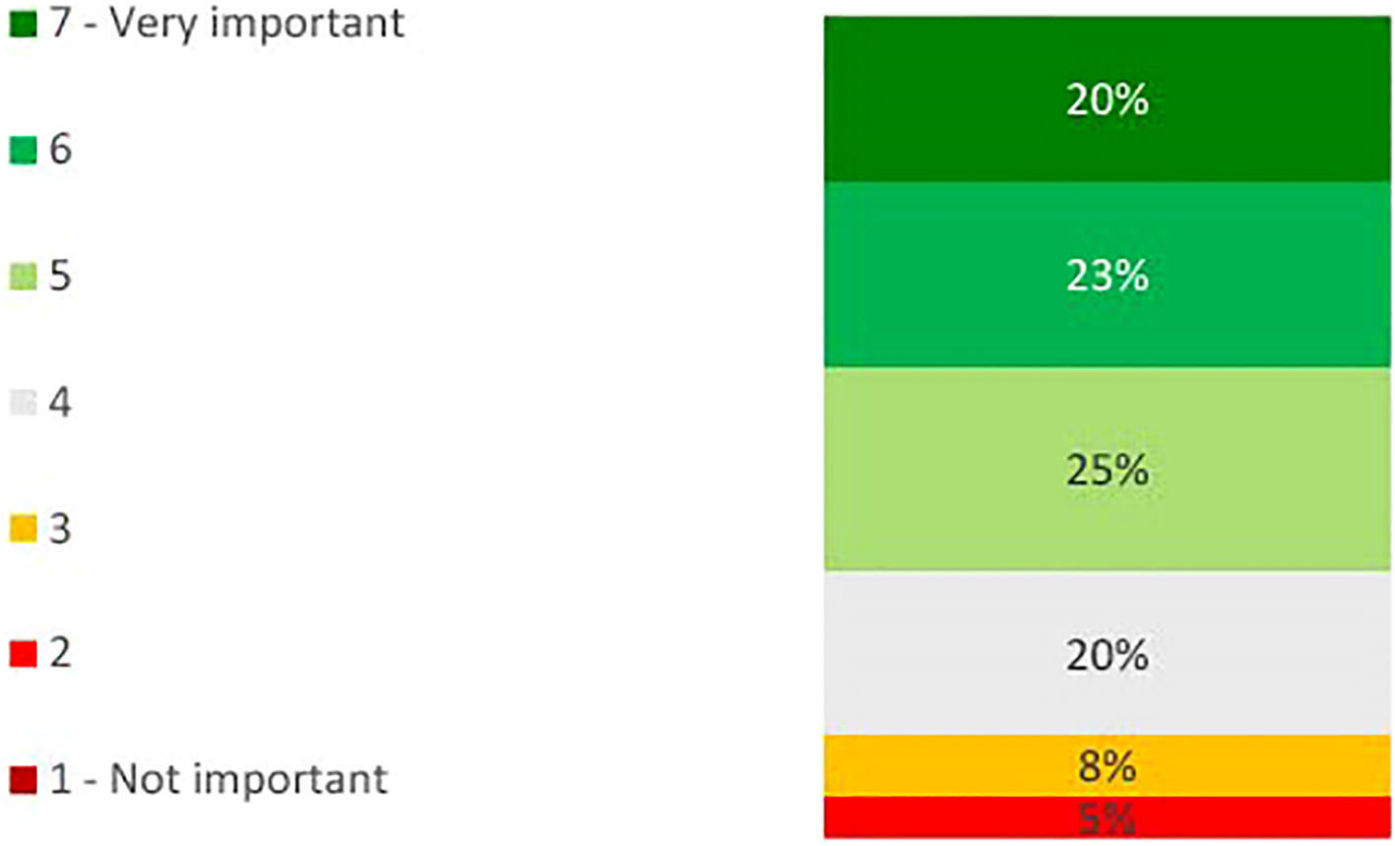
Bar chart showing percentage physicians indicating the level of importance of desensitizing patients post-transplant on a scale of 1 to 7, where 1 is ‘not at all important’ and 7 is ‘very important’ (n=40).

Physicians had mixed views on the importance of prophylactic desensitization of kidney transplant recipients post-transplant (see **Figure 5**). On a scale of 1 to 7, where 1 is not important and 7 is very important, 43% gave a score of 6 or 7. 40% use prophylactic desensitization post-transplant in some, if not all cases.

**Figure 6:**
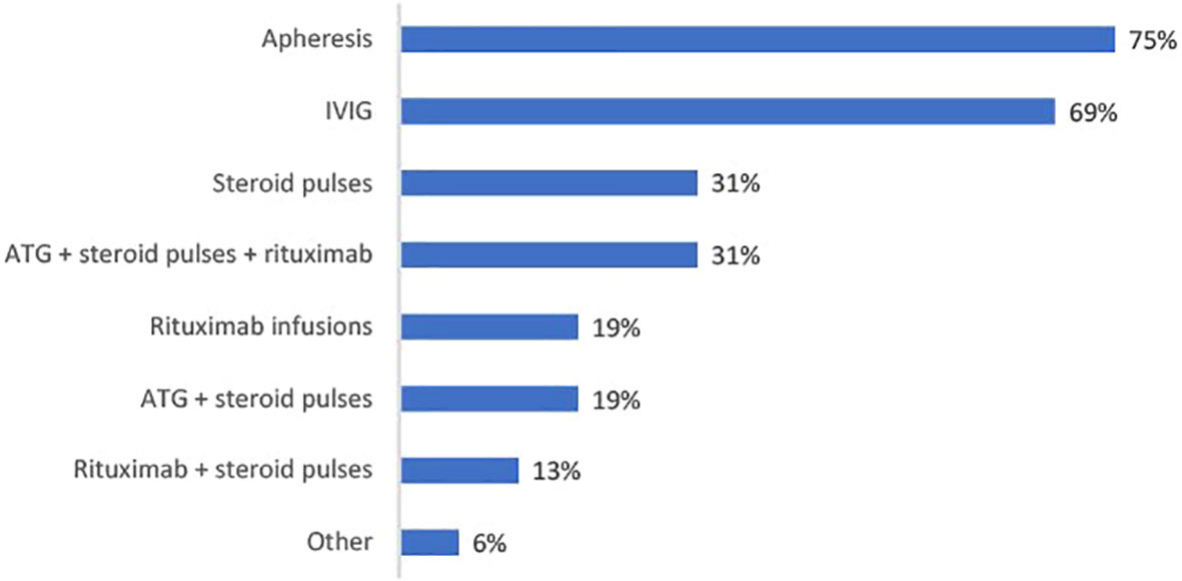
Bar chart showing percentage physicians using each prophylactic therapy as part of their post-transplant desensitization protocol (n=16).

A range of therapies are used as part of prophylactic desensitization post-transplant (See **Figure 6**). Apheresis and IVIG (75% and 69%) are the predominant therapies used among those that are desensitizing post-transplant, while steroid pulses are used by nearly a third (31%).

## Discussion

### Sensitization among kidney transplant candidates

Historically, CDC and FCM crossmatch testing were the standard measures for determining sensitization and the presence of donor-specific antibodies (DSAs). However, antibodies that are clinically irrelevant to non-HLA structures can interfere with results of these tests, leading to false positive results (10). Solid phase antibody detection assays are more sensitive in detecting anti-HLA antibodies; single bead antigen assays can identify specific HLA antibodies; therefore, they have become the current standard for anti-HLA antibody detection (14). This is reflected in the present study by the high proportion of physicians (88%) using Luminex^®^ platform assays to detect anti-HLA antibodies in potential kidney transplant recipients.

While Luminex^®^ platform assays offer greater sensitivity compared to CDC and FCM crossmatching, there are challenges in interpreting the results. Results can be used to determine the cPRA% using online calculators (15), however there is a lack of consensus on what the threshold MFI values for anti-HLA antibodies to be considered significant should be, and which antigens are considered unacceptable (16). Centers are setting their own cut-offs and may also consider other factors (in addition to MFI values) such as transplant history and specific CDC/FCM crossmatch results when determining the level of sensitization and thus the need for desensitization. It is also worth investigating the MFI threshold at which transplantations can go ahead as well as just the threshold for significance, as this may vary by center/protocol. The Sensitization in Transplantation: Assessment of Risk (STAR) meeting group suggest an MFI threshold of 1000–1500 MFI (17), which is echoed in the recent EU consensus guidelines (10). Our survey findings point to a high degree of variation in what physicians consider to be the threshold MFI level for anti-HLA antibodies to be significant. This finding further highlights the need for clarification regarding MFI thresholds that are indicative of significant HLA-antibodies and the need for desensitization. While MFI is a valuable tool, it is a semi-quantitative measure that reflects the relative strength of the antibody-antigen interaction, but does not provide an exact concentration of antibodies. Furthermore, at very high antibody concentrations the prozone effect can occur, where MFI values are underestimated due to saturation of the beads, leading to false-negative results. Factors such as complement interference, sample handling, and instrument calibration can affect MFI readings, potentially leading to inconsistent results.

The proportion of highly sensitized patients (cPRA >80%) on transplant waiting lists reported by physicians in this study is consistent, albeit slightly lower than reported in the literature (1, 9). While kidney exchange programs and optimized kidney allocation schemes have increased transplant rates in sensitized patients, not all highly sensitized patients benefit. For example, in the US, the kidney allocation scheme was implemented in 2014 to prioritize highly sensitized patients (cPRA >80%) for receiving a deceased donor kidney; this increased the rate of transplant for those with a cPRA of 99-100%, but the rate declined for patients with a cPRA of 80-94% (18). Another US study identified that despite kidney exchange and kidney allocation programs, patients with a cPRA ≥99.9% continue to have low transplant rates and high waiting list mortality rates (19). This highlights that there is, in absence of a better alternative, a need for desensitization to facilitate successful transplantation in at least a proportion of highly sensitized patients.

The important role of desensitization has not necessarily been acknowledged by all physicians. As previously mentioned, enthusiasm for desensitization has been declining over recent years, and this is reflected in that only three-fifths of physicians surveyed consider desensitization to be important.

### Desensitization protocols

The primary goals of desensitization include: reducing/removing circulating anti-HLA antibody, immunomodulation of the donor recipient’s immune system and depletion of B-cell populations to prevent rebound of DSAs that could potentially facilitate acute rejection.

Removal of anti-HLA DSAs is the primary goal, for which there is evidence to support use of apheresis pre-transplantation (20). EU guidelines recommend pre-transplant apheresis (10) and our survey findings demonstrate that most physicians prescribe it (most frequently plasmapheresis).

IVIG treatment has an immunomodulatory function and its use alone or with apheresis is well supported in the literature and guidelines (10, 20, 21). The present survey indicates that most EU physicians are using IVIG as part of their desensitization protocol.

Prevention of rebound DSAs is achievable through the use of B-cell targeting agents. Current literature supports the usage of CD-20 targeting therapies such rituximab (13, 22), and our current survey findings indicate a role for rituximab (as either a monotherapy or in combination with other treatments) as part of post-transplant desensitization.

While there is some evidence showing that post-transplant desensitization can abrogate the development of acute rejection (23), not all of the physicians surveyed considered the practice to be important. In addition to rituximab, apheresis (79%) and IVIG (69%) are regimens used by those performing post-transplant desensitization.

Several therapies may be prescribed in addition to those outlined above. Prophylactic therapies are being used by physicians when patients are undergoing pre-transplant prophylaxis. There is good rationale for this, considering there are increases in serious infection risk when using additional immunosuppression. Although this practice is not detailed in EU guidelines, our findings show that PCP prophylaxis is prescribed by the majority of physicians to those undergoing desensitization.

Induction therapies, specifically ATG and basiliximab, may be used to further reduce the risk of acute rejection (24, 25). In the present findings, while the majority of physicians surveyed are using both agents, a greater proportion are using ATG, and this is in line with evidence that suggests greater efficacy in high-risk patients (26).

Evidence from the literature supports the notion that differences in desensitization protocols can have clinical implications. For example, agents such as bortezomib (a proteasome inhibitor) have been used in combination with plasmapheresis and IVIG, but their success rates are less well-documented, with studies suggesting limited efficacy in reducing DSAs compared to rituximab-based protocols (32). However, direct comparisons between desensitization protocols are limited, and further studies are needed to ascertain survival rates and establish the optimal approach for different clinical scenarios. Furthermore, it is worth investigating whether the access to desensitization protocols are the same in all EU countries, as this would be a key contributor to the lack of consensus. The variability in clinical practice likely reflects differences in patient populations, resource availability, evidence of protocol success, and physician preferences. While the European Society for Organ Transplantation guidelines recommend the use of apheresis and IVIG as part of desensitization protocols, they acknowledge that the choice of additional agents, such as rituximab or proteasome inhibitors, may depend on individual patient factors and center experience (10). With further studies to directly compare different desensitization protocols, more robust recommendations can then be formed. To achieve this, multicenter studies comparing the success rates of different protocols, as well as the clinical rationale behind their use and success rates, are required. This would provide a stronger evidence base for developing consensus guidelines that balance efficacy, safety, and resource utilization.

### The future of desensitization

The present findings suggest that not all physicians ascribe importance to desensitization, but a range of new treatments may reignite interest in the protocol.

Imlifidase is a cysteine protease approved in the EU for the desensitization of highly sensitized adult kidney transplant patients with a positive crossmatch against an available deceased donor kidney. Given before transplantation, imlifidase is an IgG degrading enzyme that indiscriminately cleaves all classes of IgG within 6 hours, preventing formation of intact IgG for up to 7 days (27, 28). Although production of anti-HLA antibodies rebounds after duration of action, use of IVIG and rituximab following transplantation dampens this effect (27, 28). Equivalent safety and efficacy outcomes to other desensitization protocols were demonstrated through 3-year follow up data, thus providing another effective option to remove DSAs and facilitate transplantation in highly sensitized patients (29).

Obinutuzumab, a CD-20 targeting monoclonal antibody, is another emerging treatment option due to its B-cell depleting mechanism of action. It has demonstrated effective B-cell depletion in both secondary lymphoid organs and peripheral blood with evidence suggesting a longer duration of action on memory B-cells compared to rituximab (30). However, reductions in anti-HLA antibodies and the calculated panel reactive antibody score assessed using single-antigen bead assay were limited and not clinically meaningful for most patients (30). Finally, Obinutuzumab does not interfere with B-cell crossmatch, whereas rituximab does (31).

Carfilzomib (a second-generation irreversible proteasome inhibitor) has shown a reduction in MFI when used in combination with plasma exchange in a small number of patients (32). Due to its more favorable safety profile, it may offer some advantage over bortezomib (1).

The use of anti-CD38 in kidney transplant desensitization has shown promise in recent studies. CD38 is a surface protein highly expressed on plasma cells, which are responsible for producing alloantibodies and donor-specific antibodies (DSAs). Targeting CD38 with monoclonal antibodies aims to deplete these plasma cells and reduce antibody levels, facilitating transplantation in highly sensitized patients (41). An open-label, single-arm phase 1/2 trial on the anti-CD38 isatuximab demonstrated it was well tolerated and led to significant reductions in HLA-specific IgG-producing memory B cells and anti-HLA antibodies (41).

Immunomodulation and the manipulation of cytokines implicated in B-cell activation are of interest in desensitization. The monoclonal antibody tocilizumab, an anti-IL6 receptor, has demonstrated promising results when used in combination with IVIG in a phase I/II trial in those who had failed desensitization with IVIG and rituximab (33, 34). However further trials are required to determine utility in this area. A systematic review by Weinhard et al. (2021) (34) and a recent pilot study (TETRA study) by Jouve et al. (2023) (35) concluded that while IL-6 targeting does not seem to significantly improve kidney-allograft access compared to current protocols, it could improve the long-term prognosis of HLA-incompatible transplantation by hindering B-cell maturation, thereby preventing DSA rebound post-transplantation.

With a growing arsenal of therapies available for desensitization, improved protocols could allow for more highly sensitized patients to receive kidney transplants. Consequently, the time on dialysis and waitlist mortality would be reduced, leading to more successful outcomes for highly sensitized patients.

To enable robust analyses and comparisons of desensitization protocols, future studies should prioritize the collection of standardized data. Key data points should include graft survival rates, patient survival outcomes, and the incidence of antibody-mediated rejection. Additionally, longitudinal tracking of MFI levels before desensitization, immediately post-desensitization, and at regular intervals post-transplant would provide critical insights into the efficacy of different protocols. Other metrics include infection rates, adverse events related to desensitization therapies, and the proportion of patients who successfully proceed to transplantation following desensitization. Standardizing these data points across centers and studies would facilitate comparisons, improve the evidence base, and guide the development of consensus guidelines for managing highly sensitized patients.

### Study limitations

A limitation of the present study is the small number of patients who underwent desensitization prior- and post-transplants. There is also a low proportion of responding physicians based in high volume centers. Fewer patients are treated at low volume centers, and physicians employed there may have less experience in treating this patient type. Subsequently, findings may not be generalizable to how the majority of HLA-sensitized patients are treated.

Current practices within countries may not be accurately represented due to the small number of physicians that responded from each country; however, the inclusion of respondents from 20 European countries provides an understanding of attitudes towards desensitization across the continent.

This study focused on MFI due to general practice in Europe, however other aspects of desensitization such as the type of pre-DSA (A/B/DR/DQ) and flow cytometry crossmatch are other important considerations.

Another limitation of this study is the lack of follow-up data post-transplant, including graft survival rates and correlations between transplant success and MFI levels before and after desensitization. While the primary objective of this research was to assess current desensitization practices across Europe, future studies should focus on collecting longitudinal, multicenter data to evaluate the long-term outcomes of different desensitization protocols.

A further limitation of this study is the lack of detailed data on the number of highly sensitized patients who underwent desensitization, the proportion of desensitized patients who proceeded to kidney transplantation, and the breakdown of deceased versus living donors among those who were desensitized. Future studies should aim to collect and analyze these data to better understand the relationship between desensitization, donor type, and transplant outcomes.

## Conclusion

There is an ongoing need for desensitization for patients with high levels of sensitization and those that are not served by KPD/KAS programs. Recently, consensus guidelines for desensitization have been published, and while findings from this study show alignment on the usage of apheresis and IVIG, there is variability in the usage of other agents. In addition to agreement on desensitization protocols, there is also a need to educate physicians on the importance of desensitization. New therapies for desensitization are emerging and could reignite interest in desensitization, removing immunological barriers to transplantation for the most highly sensitized patients.

## Data Availability

The raw data supporting the conclusions of this article will be made available by the authors, without undue reservation.
