# Supplementary figures and images for: Use of HLA desensitization in the management of renal transplant recipients in Europe
Source: Front Immunol. 2025 Aug 28;16:1451135. doi: 10.3389/fimmu.2025.1451135 (PMC12424529; doi:10.3389/fimmu.2025.1451135)

## Supplementary material

**Figure 1:** CONSORT diagram

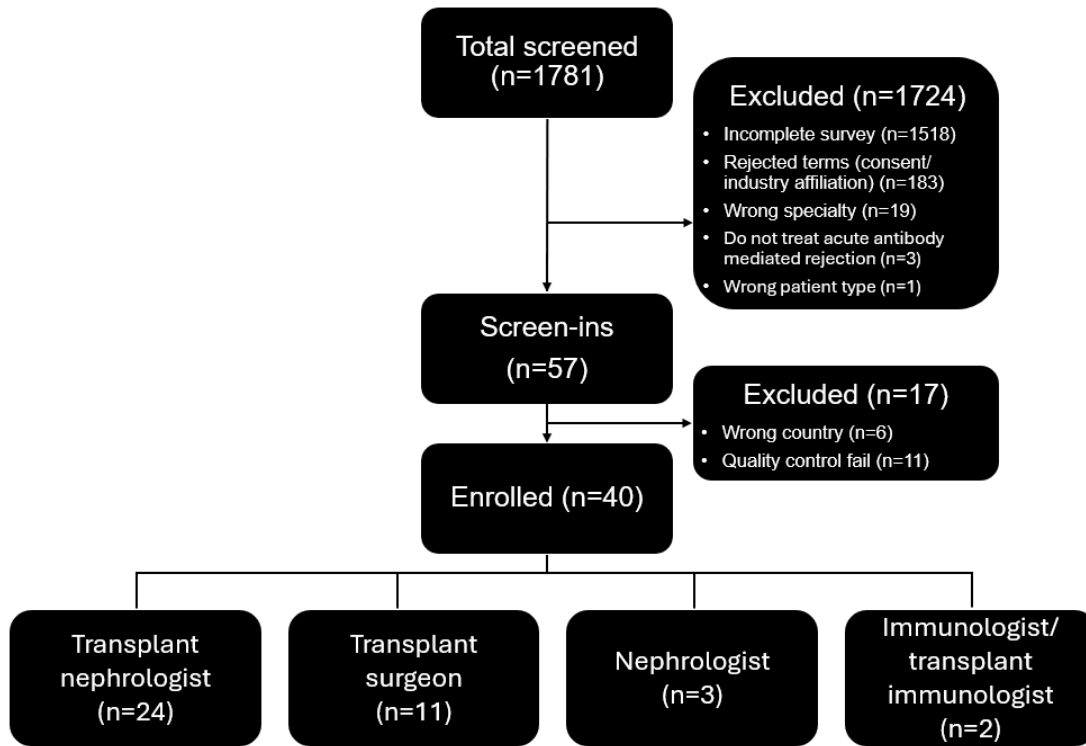

Supplement: Supplementary file 1 [file DataSheet1.pdf]
